# Dopamine production in *Enterococcus faecium*: A microbial endocrinology-based mechanism for the selection of probiotics based on neurochemical-producing potential

**DOI:** 10.1371/journal.pone.0207038

**Published:** 2018-11-28

**Authors:** Daniel Villageliú, Mark Lyte

**Affiliations:** 1 Department of Veterinary Microbiology and Preventive Medicine, College of Veterinary Medicine, Iowa State University, Ames, IA, United States of America; 2 Iowa State Interdepartmental Microbiology Program, College of Veterinary Medicine, Iowa State University, Ames, IA, United States of America; National Institute of Science Education and Research, INDIA

## Abstract

The mechanisms by which probiotics may influence host physiology are still incompletely understood. Microbial endocrinology, a field representing the union of microbiology, endocrinology and neurobiology, has theorized that microorganisms have the capacity to serve as neurochemical delivery vehicles [1]. According to microbial endocrinology, neurochemicals can serve as a common language between host and bacterium, enabling bidirectional communication. We report herein the first demonstration that *Enterococcus sp*. has the capacity to produce dopamine in a gastrointestinal-like environment when supplied with the dopamine precursor L-3,4 dihydroxyphenylalanine (L-dopa). The results presented herein provide a means to select probiotics based on neurochemical-producing potential and suggest the possibility that probiotics containing *E*. *faecium* may serve to influence the host through dopaminergic pathways.

## Introduction

*Enterococci*, including *E*. *faecium and E*. *faecalis* are natural members of the gastrointestinal flora and found in fermentation products. *E*. *faecium* is capable of producing a variety of complex molecules from aromatic compounds which influence the flavor of foods to the production of bacteriocins which show antibacterial activity against human pathogens such as Listeria [2]. Resistance to gastric juice and bile salts is common amongst this group, and *Enterococci* encountered in the diet can replicate within the gastrointestinal tract.

*Enterococci* are included within veterinary and human probiotic blends and patents for probiotic strains of *E*. *faecium* advertised as relieving functional gastrointestinal symptoms were filed over twenty years ago [3]. Veterinary examples include the probiotic blend “Probios” for large animal and Fortiflora which consists only of *E*. *faecium*. Human blends include *E*. *faecium* containing SF68 and *E*. *faecalis* containing Symbioflor 1.

Benefits of colonization with *Enterococci* include the possibility, in humans, that they may modulate the immune system and increase neutrophil phagocytosis and humoral immunity [4]. Symbioflor 1 has been used in the management of recurrent, chronic sinusitis and a double-blinded study showed clinical efficacy compared to placebo [5]. *Enterococcus-*based probiotics can benefit the management of inflammatory bowel disease (IBD) [6–12]. SF68 reportedly inhibits the growth of *Escherichia coli*, *Salmonella* serovars, *Shigella* spp. and *Enterobacter* spp. in vitro [12] while other strains reportedly decrease the incidence and severity of infections by *Chlamydiae* [13] and *Salmonella minnesota* [14]. *E*. *faecium* has been utilized in the treatment and prevention of diarrhea [15]. Controlled, double-blinded clinical studies showed that the treatment of enteritis with *E*. *faecium* SF68 shortened the duration of diarrhea and time to normalization of the stool in both adults as well as children [12].

Though there are many purported benefits to using *E*. *faecium* as a probiotic, the decision to include *E*. *faecium* within probiotic blends has thus far not derived from mechanistic evidence. This contributed to the decision by the European Food Safety Authority as well as the FDA to withhold medical approval from probiotics. Thus, a more complete understanding of the mechanisms by which probiotics function is essential in order to formulate a rational clinical use for probiotics.

## Materials and methods

### Approach to neurochemical analysis in sSIM

The techniques for the preparation of sSIM and subsequent processing of microbial broth cultures for neurochemical analysis have been recently published [16]. Briefly, ground autoclavable-grade lab diet (Teklad Global Diet #2019S, Envigo, Madison, WI, USA) was autoclaved and subjected to a multiple step *in vitro* digestion consisting of salivary, gastric and small intestinal phases as has been previously described [16]. Briefly, the oral phase consisted of mixing feed into simulated salivary fluid which contained the enzyme alpha amylase and electrolytes consistent with saliva present in the oral cavity. The entirety of the contents from the oral phase was then mixed with a simulated gastric fluid which contained the enzyme pepsin. The combination of the gastric fluid and oral phase yielded a solution with a pH and electrolyte concentration ideal for pepsin activity and consistent with the physiological concentrations observed in the stomach [17]. The gastric phase was allowed to progress over two hours in a triple mix paddle blender (Boekel Scientific, Feasterville, PA, USA) before the entirety of the contents from the gastric phase were mixed with a simulated intestinal fluid to initiate the intestinal phase. Again, the combination of all phases yielded a solution with electrolyte and pH parameters consistent with the proximal small intestine with the enzymatic activity provided by pancreatin. Once mixed, the reaction again proceeded for an additional two hours in the triple mix paddle blender. Post reaction, the mixture was supplemented with hemin and then flash frozen in liquid nitrogen and degassed [16].

The material prepared by the procedure above is termed simulated small intestinal medium (sSIM) and was used as a growth medium for the strains tested herein. Post growth, samples were acidified, deproteinated and subjected to fine filtration with a molecular weight cut off filter. Material was then analyzed by High-Pressure Liquid Chromatography with electrochemical detection (HPLC-ECD). Two HPLC-ECD units were used for the quantification of neurochemicals. For the initial survey of *E*. *faecium*, the separation and quantification of neurochemicals was performed by isocratic separation on a reversed-phase column at a flow rate of 0.6 ml min^−1^ using a Dionex Ultimate 3000 HPLC system (pump ISO-3100SD, Thermo Scientific, Bannockburn, IL, USA) equipped with a refrigerated automatic sampler (model WPS-3000TSL). The electrochemical detection system included a CoulArray model 5600A coupled with an analytical cell (microdialysis cell 5014B) and a guard cell (model 5020). Data acquisition and analysis were performed using Chromeleon 7 and ESA CoulArray 3.10 HPLC Software. For the Mucuna dose response experiment, the separation and quantification of neurochemicals was performed by Ultra High Performance Liquid Chromatography with Electro-Chemical Detection (UHPLC-ECD) on a Dionex UHPLC system which consisted of a Dionex Ultimate 3000 autosampler, a Dionex Ultimate 3000 pump and a Dionex Ultimate 3000 RS electrochemical detector (Thermo Scientific, Sunnyvale, CA, USA). Electrochemical detection was achieved with a 6041RS glassy carbon electrode set to 400mV.

### Survey of E. faecium strains

Probiotic strains of *E*. *faecium* were isolated from Probios and Fortiflora using standard microbiological techniques followed by identification on Bruker microflex MALDI-TOF system (Bruker, Billerica, MA, USA). Clinical strains of *E*. *faecium* were obtained from the Iowa State College of Veterinary Medicine Diagnostic Laboratory. All isolates had highly reliable identification by MALDI, with scores of >2.4. Strains were grown anaerobically for 24 hours on TSA agar with 5% ovine blood. Following plate growth, colonies were suspended in peptone water to make standardized suspensions with an OD_600_ measurement of 0.200 (+/- 0.005). Media was supplemented with L-dopa to a concentration of 1mM which was achieved by mixing 100μL of a 0.05M L-dopa solution into a total volume of 5mL of media. The L-dopa solution was prepared by dissolving 47mg of L-dopa into 10mL of 0.1M HCl. For a sample with a total volume of 5mL, 4.8mL of sSIM was mixed with 100μL of L-dopa spiking solution and 100μL of bacterial suspension. Inoculated samples were grown for 24 hours at 37°C, anaerobically while being subjected to low speed (100rpm) magnetic stir bar mixing. Samples were then processed by a standardized approach for catecholamines [16]. All conditions were run in triplicate.

Conversion efficiency (C.E.) was calculated by the equation: (([Dopamine]final—[Dopamine]_initial_)**/** [L-dopa]_initial_) x 100.

### Dose response experiment

A diet containing 7.5% Mucuna powder (by dry weight) was prepared by mixing 4.9 grams of Mucuna powder (Transformational Foods, Santa Barbara, CA, USA) with 60 grams of feed (Teklad Global Diet #2019S, Envigo, Madison, WI, USA). This entirety of this mixture was digested per the *in vitro* digestion technique noted previously[16]. The product Mucuna media was subsequently diluted with media not containing Mucuna to create a variety of concentrations. The *E*. *faecium* strain ML1082 was grown anaerobically for 24 hours on TSA agar with 5% ovine blood. Following plate growth, colonies were suspended in peptone water to make standardized suspensions with an OD_600_ measurement of 0.200 (+/- 0.005). Inoculation was achieved by mixing 4.9mL of media with 100μL of bacterial suspension. Inoculated samples were grown for 24 hours at 37°C, anaerobically while being subjected to low speed (100rpm) magnetic stir bar mixing. Samples were then processed by a standardized approach for catecholamines [16]. All conditions were run in triplicate.

## Results and discussion

We recently demonstrated that a simulated intestinal medium produced by the *in vitro* digestion of foodstuff enables the evaluation of neurochemical production potential by constituents of the microbiome [16]. Through an established consensus method [17], one can digest food in a manner consistent with the processes of the GI tract. When adapted to digest food to produce a microbial medium, this produces a simulated small intestinal medium that allows for the *in vitro* growth of bacteria in an environment that is reflective of the small intestinal host-based milieu [16]. By using this media to survey various probiotic mixtures, we were able to elucidate biochemical functions not previously recognized, such as the capacity of *Enterococci* to convert L-dopa to dopamine.

**Fig 1:**
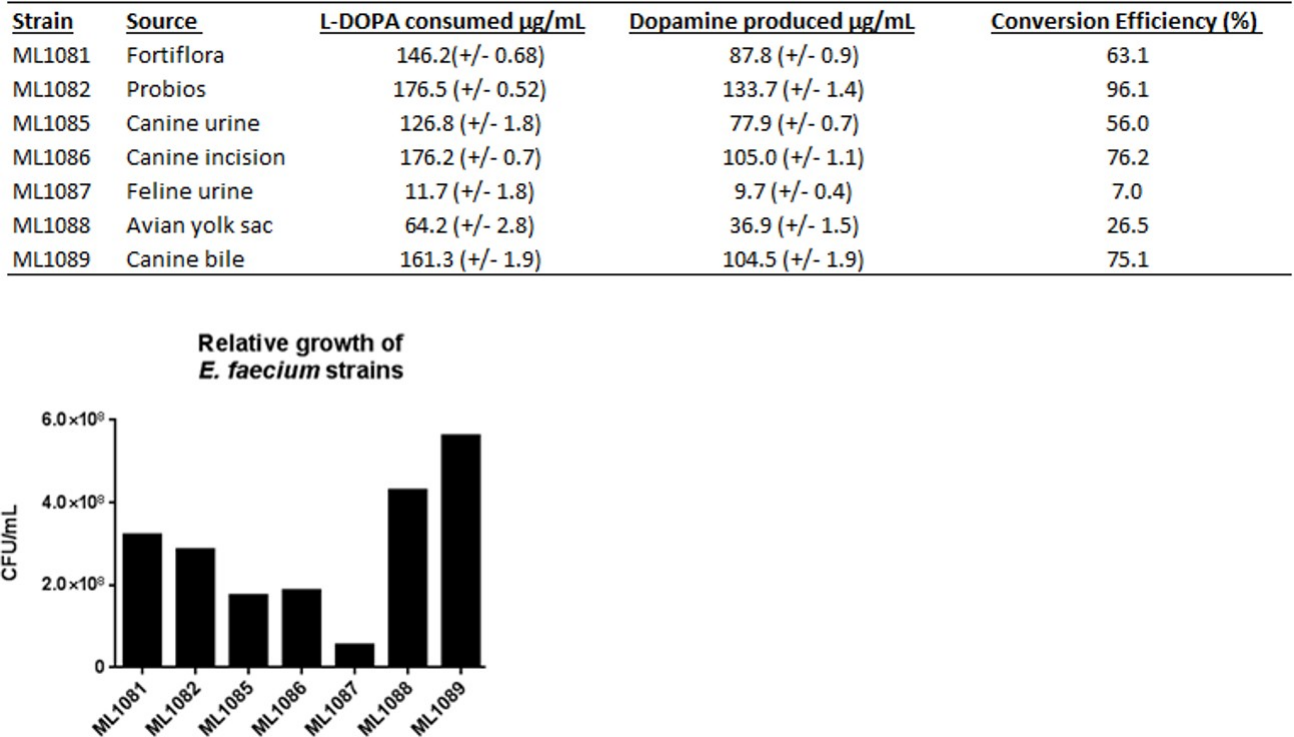
Heterogeneity in dopamine production across varying strains of *E*. *faecium*. Strains of *E*. *faecium* grown in SIM supplemented with 1mM L-dopa. (Total L-dopa available 179.1μg/mL). Top: dopamine production and L-dopa utilization efficacy. Bottom: Population differences between strains. The probiotic strain ML1082 consistently demonstrated the highest level of production at over 133 micrograms/mL. This production was more than 26% greater than the next highest producer, a clinical strain designated ML1086. Both of these strains demonstrated comparable levels of population growth and the consumption of L-dopa appeared to be exhaustive in both of these samples with less than 2% of the starting L-dopa remaining in both samples. Differences in the final dopamine concentrations of these samples appear to arise from differing efficiencies in the conversion of L-dopa to dopamine. ML1082 demonstrated a conversion efficiency of 96%, 20% higher than the efficiency of ML1086.

The strain ML1082 consistently demonstrated the highest level of dopamine production (133 μg/mL, C.E. 96%). Otherwise, there was a high level of variation in the capacity to reproduce in the gastrointestinal-like SSIM and produce dopamine (Fig 1). ML1088 demonstrated one of the highest observed levels of population growth, but was a relatively poor producer of dopamine (37 μg/mL, C.E. 27%). ML1087 produced the lowest amount of dopamine (10 μg/mL, C.E. 7%). However, this finding is attributable to the fact that ML1087 did not grow well in SSIM (5.45x10^7^ CFU/mL). All other strains were able to achieve growth on the order of 1.0x10^8^ CFU/mL with strains such as ML1089 achieving growth over a full order of magnitude greater than ML1087. Isolate-to-isolate variability in the production of neurochemicals may account for the variability in achievable clinical outcomes that are encountered with the use of different probiotic preparations [1]. The wide level of differences suggests the presence of inherent strain differences, perhaps relating to strain specific transcriptional or regulatory differences.

The present study demonstrates the hitherto unknown ability of *E*. *faecium* to produce physiologically relevant amounts of the neurochemical dopamine from dietary substrates. While it has been known for decades that probiotics, such as *Lactobacillus plantarum*, produce neurochemicals including acetylcholine [18], relatively little attention has been paid towards studying the ways the microbiota can directly modulate dopaminergic pathways. Future experiments will employ the methodology described in this report to identify *E*. *faecium* strains with demonstrated *in vitro* capacity to produce large amounts of dopamine to be administered to animals coupled with a food-based source of L-dopa such as Mucuna powder, to examine whether dopamine production occurs *in vivo*. If such experiments do, in fact, show that the administration of a synbiotic preparation of *E*. *faecium* plus an L-dopa source (such as a food-based one) leads to changes ascribable to dopaminergic pathways, then it is conceivable that dopaminergic modulation is a mechanism through which neurochemical-producing probiotics, such as *E*. *faecium*, may influence the host and modulate pathological processes such as inflammation which involve immune cells known to be responsive to neurochemicals [19].

As has been discussed in a previous report which first proposed the use of neurochemical-producing probiotics in the treatment of health and disease as well as behavior through the microbiota-gut-brain axis [1], confirming the utility of employing a neurochemical-producing probiotic *in vivo* will require carefully designed studies assessing physiological parameters of host cells grown in the presence of *E*. *faecium*. However, a review of the literature suggests that at least some of the purported effects of dopamine in the gastrointestinal tract are consistent with the effects observed when taking to *E*. *faecium*-containing probiotics. To substantiate, it is increasingly recognized that dopamine possesses an important immunoregulatory role and has the capacity to serve as a mediator enabling cross-system communication between the nervous and immune systems. Dopamine receptors are found throughout the immune system, one such example includes the demonstration that D_1_, D_2_, D_3_, D_4_ and D_5_ receptors have all been found in leukocytes [19] and it has also been shown that stimulation of D_2_ and D_3_ receptors in T cells activates T cell receptor-induced cell proliferation, and secretion of IL-2, IFN-γ and IL-4 [19]. This overlaps with some of the effects of dopamine on interleukins following the per oral administration of probiotic *E*. *faecium*. For example, it has been shown in broiler chickens that serum levels of the proinflammatory cytokines IL-1β, IL-2, IL-6 andIFN-γ, as well as the anti-inflammatory cytokines IL-4, IL-10, changed in response to the per oral administration of *E*. *faecium* strain NCIMB 11181 [20].

Given the apparent potential for both dopamine and *E*. *faecium* to modulate the immune system and promote the secretion of anti-inflammatory IL-4, it follows that both of these agents might be worth investigating for their role in the management of inflammatory diseases. In fact, *E*. *faecium* probiotics have been shown to be beneficial in the management of IBD [6–12]. Could dopamine production by *E*. *faecium* be responsible for this benefit? An association of reduced endogenous dopamine production and responsiveness with human IBD has been demonstrated [21, 22]. Further, it has been shown in an animal model of IBD induced with 2,4-dinitrofluorbenzene, that the dopamine agonist bromocriptine significantly ameliorated illness by reducing mortality, histopathologic changes such as ulceration and behavioral abnormalities such as feeding. In contrast, the dopamine antagonist domperidone significantly increased illness severity and histologic changes [23]. More recently, a study in zebra-fish demonstrated that dopamine receptor agonists alleviate enterocolitis-like inflammation whereas receptor antagonists exacerbate inflammation [24], overall it has been concluded that dopamine influences self-protective mechanisms within the gastrointestinal tract [25].

In addition to immunomodulation, dopamine has the capacity to influence intestinal motility, intestinal secretion and water absorption. In circular muscle, dopamine can induce contractions with an EC_50_ of 6.3μM. In contrast, in longitudinal muscle, dopamine can produce relaxation with an EC_50_ of 29.0μM [26]. Notably, the EC_50_ of maximal response in the distal colon was only 20.0μM which is below the 71μM production of *E*. *faecium* grown in unsupplemented media and vastly below the 1563μM *E*. *faecium* is capable of producing when L-DOPA is abundant (Table 1). Disruption of the dopaminergic transporters results in altered colonic motility [27]. Gastric epithelial cells possess dopaminergic receptors; dopamine agonists can ameliorate gastric ulcers by increasing the secretion of protective mucus and bicarbonate. Dopamine influences Na^+^, Cl^−^ and water absorption as well as K+ and bicarbonate secretion throughout various regions of the GI tract [28] and dopamine also stimulates water absorption *in vivo* [29]. There are multiple studies which have found that *E*. *faecium* can reduce the occurrence of diarrhea in animals [30–32]. Because dopamine influences the channels which uptake sodium and water from the lumen, thereby decreasing luminal water content, the production of dopamine by *E*. *faecium* may be a mechanism by which probiotics influence the water content of gastrointestinal material and alleviate diarrhea.

**Table 1:** Dopamine production increases with increasing L-dopa availability.

Growth Media	L-dopa available (μM)	L-dopa utilized (μM)	Dopamine produced (μM)
Diet with 7.5% mucuna	2172	2143	1563
Diet with 1.5% mucuna	497	489	358
Diet with 0.15% mucuna	143	142	101
Unsupplemented media	90	89	71
Media with 1mM L-dopa	934	926	721

*In vivo*, gastrointestinal L-dopa availability is likely influenced by intestinal absorption and dietary factors [33]. We thus sought to quantify dopamine production within the simulated digests of diets that contained variable amounts of a natural source of L-dopa, the legume *Mucuna pruriens* (Table 1). In a diet derived solely from lab feed, L-dopa availability permits the production of approximately 70uM of dopamine by the strain ML1082. When the dry weight of the feed digested consisted of 0.15% Mucuna, dopamine production reached over 100μM, a diet of 1.5% Mucuna allowed dopamine production in excess of 350μM and dopamine production reached 1500 μM with a diet of 7.5% Mucuna. Mucuna powder is 3% L-dopa by mass and for comparison, L-dopa rich foods like the green pods of *Vicia faba* cv Alameda can reach L-dopa concentrations as high as 6.75% of the total dry mass [34]. The generation of dopamine by *E*. *faecium* closely mirrored the availability of L-dopa. Though L-dopa is common in many raw plants and plant derived foodstuffs, L-dopa can degrade into various quinones under alkaline and thermal conditions[35]. This suggests that dietary considerations, like the processing of food or the natural presence of neurochemical precursors, may be relevant to probiotic function.

**Fig 2:**
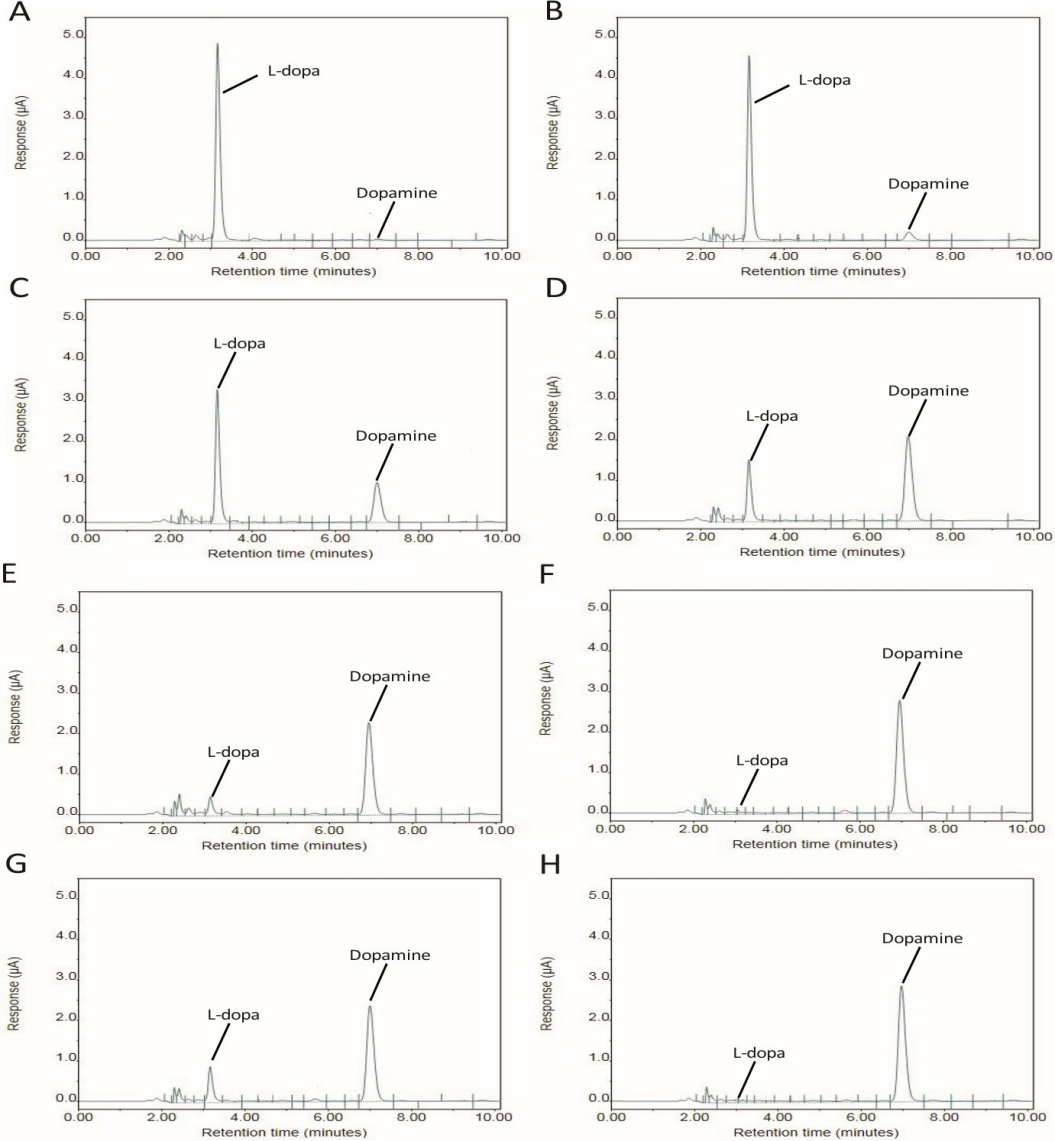
Chromatographic comparison of *E*. *faecium* strains. A) Control—No conversion. B,C) ML1087, ML1088 –Slight conversion (<30%). D) ML1085 –Moderate conversion (30%-74%). E,F) ML1089, ML1086 respectively–High level conversion (>75%). G,H) ML1081, ML1082—Two differing probiotic strains both demonstrating high levels of conversion.

When considering the efficacy of a given probiotic, one must consider the specific strain behaviors. Of high importance with *Enterococci*, would be the concern that a particular strain contains virulence traits or antibiotic resistance elements like vancomycin resistance. While these concerns are warranted, *Enterococci* are ubiquitous in the environment and these concerns should not preclude the use of an appropriately tested strain or prevent the search for other bacteria which may possess the capacity for dopamine production. The capacity to produce dopamine from L-dopa was common in all of our viable tested strains of *E*. *faecium* and may be for other species as well. Though the capacity to produce dopamine appears to be common trait in *E*. *faecium*, the conversion efficiency of L-dopa to dopamine varied greatly among isolates (Fig 2). This implies that not all strains of *E*. *faecium* are equally suitable as probiotics.

We acknowledge that a drawback of the present work is that it is relies predominantly on the behavior of a single species isolates grown *in vitro*. No matter how complex the simulated media, the dynamic environmental changes that occur in a complex microbiota constantly regulated by the host cannot be fully replicated with this approach. In a polymicrobial community we may expect that some resources would be depleted, whereas others could be liberated. We may also expect that some metabolic processes could be regulated by the presence of signaling factors inherent to the *in vivo* environment. These limitations necessitate *in vivo* follow up in order to determine the physiological significance of dopamine production by *Enterococci*. Limitations aside, sSIM media does allow one to provide microorganisms with an environment containing resources consistent with those made available from a host’s dietary intake and non-microbial processing. The *E*. *faecium* isolates tested here come from a variety of animals and anatomical sites; they demonstrate a wide range in the capacity to produce dopamine. This work makes it apparent that *Enterococci* possess the enzymatic capacity to produce dopamine and that they can do so with resources derived from the diet. Of note, *E*. *faecium* did not appear to produce dopamine from tyrosine but only from dietary sources of L-dopa. In sSIM tyrosine is regularly available in millimolar quantities [16]. However, in our research this did not appear sufficient to enable the production of significant quantities of either dopamine or L-dopa (data not shown). *Enterococci* are well known for their capacity to convert tyrosine to tyramine because of the enzyme tyrosine decarboxylase [36] and our previous work suggests that the majority of tyrosine made directly available to these strains of *E*. *faecium* is converted to tyramine [16]. *In vivo* it is conceivable that in addition to dietary sources of L-dopa, L-dopa produced by other microbes may facilitate dopamine production.

Dietary variations in L-dopa availability and the presence of other potential commensal microorganisms which can deliver L-dopa may provide a rationale for the varied response individuals taking probiotics achieve. It is conceivable that in order to achieve a benefit with *E*. *faecium*, one must also make dietary provision of L-dopa, effectively creating a synbiotic. Together these findings suggest that probiotics, like classic pharmacological interventions, may require standardized formulations and delivery routines in order to provide optimal benefits. The results presented herein demonstrate that utilization of a microbial endocrinology-based method may therefore provide for mechanistic driven approaches to identify probiotics that may be useful to treat human disease where neurochemical pathways are known to play a role. Moving forward, studies assessing the ability of *E*. *faecium* ability to modulate host tissues should also consider the availability of L-dopa and consider measuring physiological and immunological parameters known to be associated with dopaminergic regulation.
